# Evaluating Rice Bran Oil as a Dietary Energy Source on Production Performance, Nutritional Properties and Fatty Acid Deposition of Breast Meat in Broiler Chickens

**DOI:** 10.3390/foods12020366

**Published:** 2023-01-12

**Authors:** Abdulaziz Al-Abdullatif, Elsayed Hussein, Gamaleldin Suliman, Musab Akasha, Mohammed Al-Badwi, Hatem Ali, Mahmoud Azzam

**Affiliations:** 1 Animal Production Department, College of Food and Agriculture Sciences, King Saud University, Riyadh 11451, Saudi Arabia; 2 Food Science and Nutrition Department, College of Food and Agriculture Sciences, King Saud University, Riyadh 11451, Saudi Arabia

**Keywords:** chickens, palm oil, rice bran oil, meat quality, n-3 PUFA

## Abstract

The effects of rice bran oil (RBO) as an alternative dietary energy source on nutritional properties and fatty acid deposition in broiler chickens are scarce in the literature. One-day-old chickens (broiler Ross 308) were assigned in a completely randomized design with three treatment diets and nine replicates of four chickens per replicate. A basal control diet contained 4~5% palm oil (T1) in the starter and finisher phases, respectively. Treatments 2 to 3 were fed diets formulated with 50% (T2) and 100% (T3) of RBO as a fat source instead of palm oil (PO). Replacing dietary PO with RBO improved the feed conversion ratio (FCR) by 6% (*p* = 0.017) over the total period of the experiment (1–38 d of age). The feeding of RBO (T3) showed the highest (*p* < 0.001) cooking loss values in the breast meat. However, compared with other groups, the blend of PO and RBO group (T2) displayed a lower cooking loss value in the thigh meat. In breast meat, the protein content was lowered (*p* = 0.007), while the fat content was higher (*p* < 0.001) in male broiler chickens fed dietary RBO (T2 and T3). Total inclusion of dietary RBO (T3) decreased (*p* = 0.034) the proportion of saturated fatty acids (ΣSFAs) but increased (*p* = 0.02) linoleic acid. In addition, α-linolenic acid (ALA) increased (*p* < 0.001) in male broiler chickens fed dietary RBO (T2 and T3), and the highest deposit level occurred by the total inclusion of RBO (T3). Total omega 3 fatty acids (∑n-3) increased (*p* = 0.013), while the ratio of n-6 to n-3 polyunsaturated fatty acid (PUFA) decreased (*p* = 0.046) in male broiler chickens fed dietary RBO (T3) as compared with the control diet (PO; T1). In conclusion, compared with dietary PO (control diet, T1), the total inclusion of dietary RBO at 50 kg/metric ton feed (T3) increased ∑n-3, ALA, and reduced n-6:n-3 PUFA ratio in the breast meat, but cooking loss values were larger in breast and thigh meats. The blend of dietary PO and RBO (T2) was better for both production performance targets (feed intake and FCR), cooking loss values, and deposition of ALA in the breast meat. The inclusion of dietary RBO into broiler diets needs further study, but the present experiment aids in expanding research knowledge to make that possible.

## 1. Introduction

Dietary lipids derived from plant and animal sources are used in the formulation of broiler feeds. The inclusion of 2~5% dietary lipids is recommended by nutritionists to support rapid growth and improve the deposition of fatty acids in the muscles [1,2,3]. Palm oil (PO) is a by-product of processing the fruit of the oil palms. It is routinely used in human and animal nutrition, and thus, is considered the most remarkable global oil crop, supplying about 40% of global vegetable oil consumption [4]. PO contains concentrations of saturated fatty acids (SFAs) and it is distinguished by its high concentrations of palmitic acid (C16:0). In addition, it contains antioxidant components such as β-carotene, coenzyme Q10, and polyphenols [5]. It has been concluded that PO has favorable results on the firmness of chicken meat as compared with dietary lipids rich in unsaturated fatty acids [6].

Finding alternative fat sources is a favorable action for sustainable broiler production. In this regard, RBO is a by-product of processing rice; it is extracted from the germ and inner husk. The oil level in rice bran ranges between 10% and 23% [7]. The fatty acid proportion of RBO comprises 41% monounsaturated (MUFA), 36% polyunsaturated (PUFA), and 19% SFA [8], thus, it is a near-ideal fatty acid composition that could be used to produce functional foods. In addition, RBO is considered an acceptable vegetable oil due to its bioactive compounds and its cardioprotective potential [9]. The unsaponifiable lipids of RBO have great nutritional value; it is enriched with gamma (γ) oryzanol and tocotrienols and tocopherols that have antioxidant properties [10]. Previous studies found that RBO can be used in broiler and pig diets [11,12]. In addition, a recent study has found that dietary RBO stimulated the growth of broiler chickens due to greater digestibility of ether extract, fatty acids, and apparent metabolizable energy than PO [13]. It is well known that PUFAs have beneficial roles in cardiovascular system disorders in humans, and the enrichment of chicken meat with omega-3 fatty acids provides a functional food source. At the same time, high levels of PUFA in chicken meat increase the oxidation process and negatively affect smell, taste, nutritional value, and shelf-life [14]. The nutritional modulation strategy to enhance the meat quality of broiler chickens is of worldwide research interest because chicken meat is safe and acceptable [15,16,17,18]. In addition, it has been found that combinations of dietary lipids sources are more beneficial than a single fat source and promote the growth and meat quality of broiler chickens together with enrichment in essential fatty acids of breast meat [19,20,21,22]. Owing to the above reasons, we hypothesized that RBO as a single fat source or a combination of RBO and PO in broiler diets may improve production performance, nutritional properties, and fatty acid deposition in the breast meat in broiler chickens. The effects of RBO on nutritional properties and fatty acid deposition in the breast meat of broiler chickens are scarce in the literature. Therefore, the goal of our study was to evaluate rice bran oil as an alternative dietary energy source on growth performance, processing yields, meat quality, chemical composition, and fatty acid deposition in the breast meat in broiler chickens

## 2. Materials and Methods

### 2.1. Experimental Design and Diets

One-day-old chickens (broiler Ross 308) were vaccinated for Marek’s disease, Newcastle disease, and Infectious bronchitis (IB) at the hatchery and were moved to our research unit. They were housed in wire battery cages (58 cm × 50 cm × 35 cm) and the cages were equipped with a heating system, nipple drinkers, and feed troughs. From d 1 to 7 of age, the initial brooding temperature was set at 32 ± 1 °C and was lowered gradually to 24 ± 1 °C on d 24 of age. The experimental protocol was approved by the Animal Care and Use Committee of King Saud University, Riyadh, Saudi Arabia (KSU-SE-20-74) according to the Guide for the Care and Use of Agricultural Animals in Research and Training [23].

Chickens were assigned in a completely randomized design with three treatment diets and nine replicates of four chickens per replicate. The experimental treatments consisted of diets formulated based on corn-soybean meal. A basal control diet contained 4~5% palm oil (T1) in the starter and finisher phases, respectively. Treatments 2 to 3 were fed diets formulated with 50% (T2) and 100% (T3) of rice bran oil (RBO) as a fat source instead of palm oil (PO). The chickens were fed mash diets with ad libitum access to feed and water. Feedstuff ingredients and nutrient compositions of the experimental diets are shown in Table 1. Experimental diets were analyzed using [24] according to [25] to determine the proximate analysis of feeds. In preparation for the analysis of the fatty acid composition, each sample was homogenized (3 × 10 s at 3000 rpm) with chloroform/methanol mixture (2:1, *v*/*v*) to an eventual volume of 45 mL. The derived supernatant was used for the preparation of fatty acid methyl esters (FAME)using a mixture of methanol/sulfuric acid (95:5) and hexane following the method proposed by [26]. Hexane extract obtained from FAMEs was used for the quantification of free fatty acids using an Agilent 7890A GC/FID system (Agilent Technologies, Palo Alto, CA, USA) equipped with a column DB-23 (60 m × 0.25 mm × 0.25 μm) to determine the FAME. The FAME peaks were analyzed by comparing the retention times of the standard fatty acid mixture (Cat. No. 24073, Sigma-Aldrich, St. Louis, MO, USA) using the Hewlett-Packard ChemStation software (Agilent Technologies Inc., Wilmington, DE, USA). Fatty acid contents are shown in Table 2.

### 2.2. Growth Performance Data

Initial body weights were recorded on a cage basis. Body weights and feed intake were measured at 21 d and 38 d of age per replicate. FCR was calculated as feed intake per body weight gain (g/g).

### 2.3. Carcass Traits

Nine male chickens with similar body weights were selected per treatment (one chicken per replicate) to evaluate carcass traits as described by [27]. Chickens were slaughtered as proposed in [25] and each carcass was de-feathered and autopsied. The weights of fat pads and hot carcasses were measured immediately after evisceration, then they were placed in a refrigerator (4 °C; 24 h) for cooling. Cold carcasses were weighed and cut into main parts to determine the absolute weights and relative weights of the breast, legs, and wings (g/100 g).

### 2.4. Meat Quality

The *Pectoralis major* from the left side of the breast meat, as well as thigh meat samples without skin, were individually packed in bags (PA/PE, 90 μm), evacuated using a Komet Plus Vac 20 Vacuum Sealer (KOMET Maschinenfabrik GMBH Am Filswehr 1, D-73207 Plochingen, Germany), and frozen at −20 °C until further use. Briefly, initial and ultimate pH values were measured using a portable pH meter (HI-99,163; Hanna Instruments, Woonsocket, RI, USA). To determine ultimate pH values, the meat was taken from a refrigerator and held at 24 °C (room temperature) for 30 min to allow for blooming before making the measurements. To determine the cooking loss, the frozen samples were thawed at 4 °C for 24 h at the time of analysis, and a countertop grille (FHG 43302 SS, Stainless Steel, Kalorik, Belgium) was used for cooking the *Pectoralis major* and thigh muscles at 200 °C to reach an internal temperature of 70 °C as described by [28]. Cooking loss (CL) was determined by the difference between the sample’s initial and ultimate weights then dividing it by the initial weight of the same sample.

### 2.5. Nutritional Properties and Fatty-Acid Deposition of the Breast Meat Samples

From nine additional chickens per treatment, all skins were removed from the breast meat to determine the proximate analysis and fatty acids. The breast portion was homogenized with a grinder at 7000× *g* for 10 s, then vacuum packed and stored in the freezer at −20 °C until analysis. The samples were then freeze-dried and ground again using a Panasonic grinder (MK-G20MR, Japan) to obtain a fine powder. The moisture (950.46), crude ash (920.153), ether extract (991.36), and crude protein (981.10) content were determined as described in [24]. In preparation for the analysis of the fatty acid composition, samples of lyophilized breast meat were ground. Each sample was homogenized (3 × 10 s at 3000 rpm) with chloroform/methanol mixture (2:1, *v*/*v*) to an eventual volume of 45 mL. The same method as for fatty acids in the diet analysis was applied as described above in Section 2.1 and values were expressed as g/100 g of breast meat.

### 2.6. Statistical Analysis

The data were analyzed using SPSS [29]. Statistically significant differences between the mean values were analyzed by a post-hoc Tukey’s test. Replicate cages (n = 9/TRT) were designated for growth performance analysis and, for samples, one broiler chicken was slaughtered per replicate. The statement of significance between means was considered at *p* < 0.05.

## 3. Results

### 3.1. Growth Performance

Growth performance data are shown in Table 3. Dietary RBO (T2 and T3) had no effect on growth performance up to 21 d of age (starter phase). However, dietary RBO improved FCR by 6% (*p* = 0.017) over the total period of the experiment (1–38 d of age).

### 3.2. Carcass Characteristics

Carcass characteristics data of male broiler chickens are presented in Table 4. Dietary treatments had no effects (*p* > 0.05) on absolute weights or yields of hot and cold carcasses, fat pads, or carcass cuts.

### 3.3. Physical Parameters of Meat

The physical parameters of the *Pectoralis major* in male chickens are presented in Table 5. Total inclusion of dietary RBO (T3) showed the highest (*p* < 0.001) cooking loss values in the breast meat (*Pectoralis major*). However, compared with other groups, the blend of PO and RBO group displayed lower cooking loss values in the thigh meat. The initial and ultimate pH decreased (*p* < 0.001) in the *Pectoralis major* and thigh meat in response to feeding with RBO (T2 and T3).

### 3.4. Nutritional Properties of the Breast Meat

The nutritional properties of the breast meat of male broiler chickens are presented in Table 6. In breast meat samples, protein content was lowered (*p* = 0.007), while fat content was higher (*p* < 0.001) in male broiler chickens fed dietary RBO (T2 and T3), with no alteration in ash contents (*p* = 0.93). The moisture content tended to be lowered by the total inclusion of dietary RBO (T3).

### 3.5. Fatty-Acid Deposition of the Breast Meat

The Fatty acid deposition of the breast meat of male broiler chickens is presented in Table 7. Total inclusion of dietary RBO (T3) decreased (*p* = 0.034) the proportion of ΣSFAs while increasing (*p* = 0.02) linoleic acid. In addition, ALA increased (*p* < 0.001) in male broiler chickens fed dietary RBO (T2 and T3), and the highest deposit level occurred with the total inclusion of dietary RBO (T3). Moreover, ∑n-3 PUFA increased (*p* = 0.013), while the ratio of n-6 to n-3 PUFA decreased (*p* = 0.046) in male broiler chickens fed total inclusion of dietary RBO (T3) as compared with the total inclusion of PO (control diet, T1), with no alteration in n-6 PUFA (*p* = 0.032) among dietary treatments.

## 4. Discussion

The world human population may reach about 9.3 billion in 2050, and about 11.2 billion in 2100 [30]. Consequently, to feed the enormous population, food production needs to be increased. Chicken meat is the primary food source of animal protein intake due to its low-fat content and low cholesterol levels [31]. Generally, there are no cultural, religious, lifestyle, or health-concern restrictions on chicken meat intake. At the same time, food consumption awareness based on health grounds is having an increasing influence on consumer food choices [32].

The positive effect of dietary RBO on FCR may be a result of the degree of unsaturation [14]. It has been found that PUFAs are easier to be oxidized for energy compared to SFAs [33]. In addition, it should be highlighted that the significant effect of RBO on FCR may be due to a reduction in feed intake. It has been reported that chickens ate more feed when diets contained palmitic or stearic acid than linoleic or oleic acid [34]. It has been found that dietary unsaturated fatty acids increased available metabolizable energy owing to their higher digestibility and absorption efficiency compared to SFAs [35]. Therefore, the improvement in FCR in the present study could be explained by the positive effect of RBO on metabolism and energy expenditure. The current findings related to the improvement of FCR are in agreement with the findings of previous studies [14,36,37]. Herein, an improvement in FCR was not observed during the starter phase (d 1–21). The current findings indicated that dietary fatty acid composition had no effect on FCR and overall growth performance data during the starter phase. This finding may be due to the lack of change in feed intake among dietary treatments during this period. Our results are in agreement with those of Ayed et al. [38] who observed increased feed intake in broiler chickens fed 3% PO from d 17 to 38 of age (finisher phase) but feed intake was unaffected from d 1 to 16 of age.

The proportions of the important carcass cuts (breast, legs, and wings), carcass yield, and fat pad did not differ due to dietary treatments. The current results indicate that experimental treatments had enough energy availability for muscle growth.

The feeding of RBO (T3) increased cooking loss values in breast meat (*Pectoralis major*) and thigh meat. It has been mentioned by [39] that unsaturated fatty acids may increase cooking loss. The high inclusion level of fat rich in PUFA elevated lipid peroxidation and tissue injuries in animals [40,41], which could cause severe protein denaturation (denaturation of sarcoplasmic proteins) and reduce the protein’s ability to bind water [42]. It is well known that CL is inversely related to WHC [43]. In addition, herein, initial and ultimate pH decreased by feeding RBO diets (T2 and T3) in both Pectorals major and thigh meat. It has been reported that low WHC and low pH resulted in high CL [44], while the higher pH_24h_ in the muscle of broiler chickens indicated a better shelf life and WHC [39,45]. It has been found that increased fat levels in pork loins increased CL [46]. In addition, it has been reported a positive correlation between lipid level and CL [47]. Herein, fat content was higher in response to feeding with dietary RBO. No previous studies presented the effect of RBO on cooking loss data of chicken meat. However, values of cooking loss herein were around 17~32%; these values are in agreement with the findings found in chicken meat [48,49]. In breast meat, the protein content was lowered, while fat content was higher in response to replacing PO partially or totally by RBO (T2 and T3). However, mean values of protein and fat contents in all experimental groups were within the reference range [50]. The reason for the reduction in protein content and increased fat content might be associated with disrupted lipid metabolism (lipolysis) resulting in more fat deposition in muscles. Lipid oxidation is one of the main mechanisms of meat deterioration [51,52]. The stage of protein oxidation could be initiated by lipid oxidation [53,54], which induces intramolecular and intermolecular polymerization of proteins and negatively affects WHC, which may explain the current results on cooking loss.

Chicken meat represents a poor source of n-3 -PUFA; however, it is possible to improve their deposition through nutritional strategies. Herein, the ratio of n-6 to n-3 PUFA in the breast meat decreased in response to feeding with total inclusion of RBO (T3). It is well known that a lower ratio of n-6/n-3 in meat is more desirable for human health [55]. The total inclusion of dietary RBO (T3) increased ∑n-3. In addition, α-linolenic acid (ALA) increased in response to feeding with RBO (T2 and T3), and the highest deposit level occurred with the total inclusion of RBO (T3), indicating that RBO has the potential to enrich food with n-3 PUFA (omega-3). There is a trend toward increasing the n-3 PUFA deposition in meat, which is essential for the health of humans (brain, retina, and cardiovascular disease). In addition, it has been found that n-3 PUFA induced fatty acid oxidation, but enhanced energy expenditure [56]. Thus, positive findings of RBO on FCR could be explained by improved metabolism and energy expenditure, while negative effects of dietary RBO on fat content and cooking loss of breast meat could be explained by increased fatty acid oxidation.

## 5. Conclusions

Compared with dietary PO (control diet, T1), the total inclusion of dietary RBO at 50 Kg/metric ton feed (T3) increased ∑n-3, ALA, and reduced the n-6:n-3 PUFA ratio in the breast meat, but cooking loss values were larger in breast and thigh meats. The blend of dietary PO and RBO (T2) was better for both production performance targets (feed intake and FCR), cooking loss values, and deposition of ALA in the breast meat. The inclusion of dietary RBO into broiler diets needs further study, but the present experiment aids in expanding research knowledge to make that possible.

## Figures and Tables

**Table 1:** Ingredients and nutrient composition of the experimental diets.

Ingredients, %	Starter Diet (d 1–21)			Finisher Diet (d 22–38)		
	PO	PO-RBO	RBO	PO	PO-RBO	RBO ^1^
	T1	T2	T3	T1	T2	T3
Yellow corn	57.40	57.40	57.40	60.00	60.00	60.00
Soybean meal, 48%	34.40	34.40	34.40	31.00	31.00	31.00
Di-calcium phosphate	1.70	1.70	1.70	1.63	1.63	1.63
Limestone	1.00	1.00	1.00	0.91	0.91	0.91
NaCl	0.50	0.50	0.50	0.47	0.47	0.47
L-Lys	0.10	0.10	0.10	0.11	0.11	0.11
Dl-Meth	0.27	0.27	0.27	0.25	0.25	0.25
L-Thr	0.03	0.03	0.03	0.03	0.03	0.03
Choline chloride	0.10	0.10	0.10	0.10	0.10	0.10
Premix ^2^	0.50	0.50	0.50	0.50	0.50	0.50
Palm oil	4.00	2.00	-^3^	5.00	2.50	-^3^
Rice bran oil ^4^	-	2.00	4.00	-^3^	2.50	5.00
Total	100	100	100	100	100	100
	Calculated nutrient level%					
Crude protein	21.30	21.30	21.30	19.00	19.00	19.00
Digestible Lys	1.12	1.12	1.12	0.99	0.99	0.99
Digestible Met + Cys	0.85	0.85	0.85	0.77	0.77	0.77
Digestible Thr	0.75	0.75	0.75	0.68	0.68	0.68
Calcium	0.91	0.91	0.91	0.83	0.83	0.83
Non-phytate P	0.44	0.44	0.44	0.42	0.42	0.42
ME, Kcal/Kg	3100	3090	3080	3190	3170	3160
	Analyzed nutrient level%					
Dry matter	92.26	91.81	92.01	91.39	91.82	91.91
Crude protein	22.2	21.75	21.3	19. 32	19.11	19.54
Ether extract	6.71	6.99	7.33	7.91	7.87	7.99
Total crude fiber	1.69	1.7	1.7	1.82	1.47	1.56
GE, Kcal/Kg	4216	4281	4258	4315	4322	4308

^1^ PO = dietary palm oil; PO-RBO = The blend of dietary palm oil and rice bran oil; RBO = dietary rice bran oil. ^2^ Premix per 5 kg: Vit. A, 2,400,000 IU; D3, 1,000,000 IU; E, 16,000 IU; K3, 800 mg; B1, 600 mg; B2, 1600 mg; B3, 8000 mg; B5, 3000 mg; B6, 1000 mg; biotin 40 mg; B9, 400 mg; B12, 6 mg; Minerals: Cu, 2000 mg; I, 400 mg; iron, 1200mg; Mn, 18,000 mg; Se, 60 mg, and Zn, 14,000 mg. ^3^ no date. ^4^ The concentrations of Vitamin E and γ-oryzanol were 33.03 mg and 614 mg/100 mL, respectively.

**Table 2:** Fatty acid content of the experimental diets.

Fatty Acids, g/100 g	Starter Diet (d 1–21)			Finisher Diet (d 22–38)		
	PO	PO-RBO	RBO	PO	PO-RBO	RBO ^1^
	T1	T2	T3	T1	T2	T3
C11:0	0.13	0.00	0.00	0.00	0.00	0.00
C12:0	0.82	0.56	1.10	0.59	0.43	1.08
C14:0	0.61	0.70	0.36	0.69	0.44	0.54
C16:0	28.37	23.50	17.08	30.39	24.24	18.37
C16:1 (n-7)	0.16	0.19	0.17	0.21	0.16	0.31
C17:0	0.00	0.31	0.00	0.17	0.10	0.28
C18:0	3.66	3.26	2.35	3.73	3.17	2.78
C18:1 (n-9)	37.05	37.89	37.72	38.70	39.04	38.34
C18:1 (n-7)	0.75	0.88	0.86	0.87	0.84	1.03
C18:2 (n-6)	26.5	29.88	37.98	22.57	29.20	33.95
C18:2 (n-4)	0.17	0.13	0.14	0.16	0.16	0.17
C18:3 (n-3)	1.02	1.19	1.4	0.78	1.04	1.26
C18:4 (n-1)	0.00	0.00	0.00	0.41	0.00	0.00
C20:0	0.43	0.56	0.76	0.52	0.63	0.87
C20:1 (n-9)	0.16	0.29	0.38	0.23	0.27	0.47
C22:0	0.18	0.14	0.24	0.15	0.29	0.54
C22:1 (n-9)	0.00	0.00	0.00	0.12	0.00	0.00
∑SFA	34.2	29.16	21.89	36.24	29.3	24.46
∑MUFA	38.12	38.96	39.13	40.13	40.31	40.15
∑n-3	1.02	1.19	1.40	0.78	1.04	1.26
∑n-6	26.5	29.88	37.98	22.57	29.20	33.95
∑PUFA	27.52	31.07	39.38	23.35	30.24	35.21
n-6:n-3	25.98	25.11	27.12	28.94	28.08	26.94
n-3:n-6	0.038	0.039	0.036	0.035	0.036	0.037
∑n-9	37.21	38.18	38.1	39.05	39.31	38.81
∑UFA	65.64	70.03	78.51	63.48	70.55	75.36
UFA:SFA	1.92	2.40	3.58	1.75	2.40	3:00

^1^ PO = dietary palm oil; PO-RBO = The blend of dietary palm oil and rice bran oil; RBO = dietary rice bran oil SFA = Saturated fatty acids; MUFA = Mono-unsaturated fatty acids; n-3 = omega 3; n-6 = omega 6; PUFA = Polyunsaturated fatty acids; ∑n-9 = omega 9; UFA = Total unsaturated fatty acids.

**Table 3:** Growth performances of broiler chickens over 38 d of age ^1&2^.

Items	PO	PO-RBO	RBO ^3^	SEM	*p*-Value
	T1	T2	T3		
d 1–21 (Starter phase)					
Initial body weight, g (d 1)	41.81	41.77	41.81	0.056	0.69
Body weight, g	837	835	826	34.40	0.94
Body weight gain, g	795	793	784	34.38	0.94
Feed intake, g/b	1006	962	958	39.34	0.41
FCR, g/g	1.265	1.213	1.222	0.029	0.16
d 22–38 (Finisher phase)					
Body weight gain, g	1511	1516	1538	75.79	0.93
Feed intake, g/b	2401 ^a^	2243 ^b^	2267 ^ab^	59.04	0.03
FCR, g/g	1.589	1.479	1.474	0.056	0.10
d 1–38 (Over growth period)					
Body weight, g	2348	2351	2364	70.146	0.97
Body weight gain, g	2306	2309	2322	70.155	0.97
Feed intake, g/b	3407 ^a^	3205 ^b^	3225 ^ab^	72.257	0.020
FCR, g/g	1.48 ^a^	1.39 ^b^	1.39 ^b^	0.033	0.017
PRC ^2^, %		−6	−6		

^1^ n = 9 replicates per treatment (4 birds/Rep.); ^2^ survivability rate was 100% for all groups; ^2^ Percentage in relation to the control = mean of TRT- mean of control diet/mean of the control diet × 100; ^3^ PO = dietary palm oil; PO-RBO = The blend of dietary palm oil and rice bran oil; RBO = dietary rice bran oil Means in a row with different letters are different (*p* < 0.05).

**Table 4:** Carcass characteristics of male broiler chickens ^1^.

Items	PO	PO-RBO	RBO ^2^	SEM	*p*-Value
	T1	T2	T3		
Absolute weights (g)					
Hot carcass ^3^	2169	2163	2039	89.30	0.27
Cold carcass	2121	2114	1992	88.03	0.27
Breast meat	663	670	623	38.19	0.42
Legs meat	552	550	547	26.61	0.98
Wings meat	147	146	145	8.16	0.97
Fat pad	31.85	31.15	31.85	5.14	0.98
Yield (g/100 g)					
Hot carcass	75.21	75.00	74.43	0.61	0.43
Cold carcass	73.53	73.28	72.71	0.58	0.37
Breast meat	22.99	23.28	22.73	0.96	0.84
Legs meat	19.15	19.07	20.00	0.56	0.21
Wings meat	5.09	5.04	5.29	0.19	0.42
Fat pad	1.11	1.07	1.15	0.17	0.91

^1^ n = 9 birds per treatment; ^2^ PO = dietary palm oil; PO-RBO = The blend of dietary palm oil and rice bran oil; RBO = dietary rice bran oil. ^3^ Excluding head, neck, feet, abdominal fat, and internal organs (expressed as % from BW).

**Table 5:** Physical parameters of *Pectoralis major* in male chickens **^1^**.

Physical Traits	PO	PO-RBO	RBO ^2^	SEM	*p*-Value
	T1	T2	T3		
*Pectoralis major*					
Cooking loss, %	17.60 ^b^	19.86 ^b^	31.30 ^a^	2.666	<0.001
Meat pH					
pH_20min_	6.51 ^a^	6.11 ^b^	6.08 ^b^	0.080	<0.001
pH_24hr_	6.15 ^a^	6.09 ^b^	6.03 ^b^	0.023	<0.001
Thigh meat					
Cooking loss, %	32.67 ^a^	23.64 ^b^	31.32 ^a^	1.724	<0.001
Meat pH					
pH_20min_	6.50 ^a^	6.25 ^b^	6.23 ^b^	0.055	<0.001
pH_24hr_	6.27 ^a^	6.15 ^b^	6.12 ^b^	0.032	<0.001

^1^ n = 9 replicates per treatment; Means in a row with different letters are different significantly (*p* < 0.05) ^2^ PO = dietary palm oil; PO-RBO = The blend of dietary palm oil and rice bran oil; RBO = dietary rice bran oil.

**Table 6:** Nutritional properties of breast meat of male chickens **^1^**.

Proximate Composition, %	PO	PO-RBO	RBO ^2^	SEM	*p*-Value
	T1	T2	T3		
Moisture	74.08	74.25	73.66	0.228	0.050
Protein	24.17 ^a^	23.55 ^b^	23.45 ^b^	0.206	0.007
Fat	0.45 ^c^	0.77 ^b^	1.63 ^a^	0.069	<0.001
Ash	1.15	1.18	1.18	0.090	0.93

^1^ n = 9 replicates per treatment; Means in a row with different letters are different significantly (*p* < 0.05); ^2^ PO = dietary palm oil; PO-RBO = The blend of dietary palm oil and rice bran oil; RBO = dietary rice bran oil.

**Table 7:** Fatty acid contents of breast meat of male chickens **^1^**.

Fatty Acids Composition, g/100 g of FAME	PO	PO-RBO	RBO ^2^	SEM	*p*-Value
	T1	T2	T3		
SFA					
Lauric acid, C12	0.33	0.39	0.56	0.11	0.19
Myristic acid, C14	0.54	0.53	0.51	0.07	0.93
Palmitic acid, C16	24.80 ^a^	23.48 ^ab^	21.96 ^b^	0.72	0.02
Heptadecanoic acid, C17	0.18	0.14	0.31	0.07	0.13
Stearic acid, C18	7.72	7.00	7.52	0.68	0.58
Arachidic acid, C20	0.14	0.13	0.17	0.01	0.21
Lignoceric acid, C24	0.22	0.19	0.24	0.05	0.71
∑SFA	33.95 ^a^	31.87 ^ab^	31.28 ^b^	0.78	0.03
MUFA					
Palmitoleic acid, [C16:1 (n-7)]	3.14	3.12	2.88	0.34	0.71
Vaccenic acid, [C18:1 (n-7)]	1.88	1.76	1.92	0.14	0.55
Oleic acid, [C18:1 (n-9)]	35.34	35.47	34.76	1.78	0.91
Gondoic acid, [C20:1 (n-9)]	0.29	0.31	0.35	0.02	0.07
∑MUFA	40.66	40.67	39.92	1.93	0.90
PUFA					
Linoleic acid, [C18:2 (n-6)]	17.35 ^b^	20.39 ^ab^	21.52 ^a^	1.10	0.02
Gamma-linolenic acid, [C18:3 (n-6)]	0.19	0.2	0.23	0.03	0.43
Eicosadienoic acid, [C20:2 (n-6)]	0.50	0.44	0.49	0.11	0.88
γ-linolenic acid [(DGLA) C20:3 (n-6)]	0.67	0.62	0.55	0.10	0.52
Arachidonic acid, [C20:4 (n-6)]	3.57	3.31	3.01	0.74	0.76
Docosatetraenoic acid, [C22:4 (n-6)]	0.98	0.80	0.82	0.21	0.64
Docosapentaenoic acid, [C22:5 (n-6)]	0.27	0.29	0.19	0.07	0.40
∑n-6	23.55	26.07	26.83	2.08	0.32
α-Linolenic acid [(ALA), C18:3 (n-3)]	0.52 ^c^	0.717 ^b^	0.86 ^a^	0.013	<0.001
Docosapentaenoic acid [(DPA), C22:5 (n-3)]	0.25	0.27	0.27	0.067	0.96
Docosahexaenoic acid [(DHA), C22:6 (n-3)]	0.20	0.17	0.2	0.028	0.44
∑n-3	0.98 ^b^	1.16 ^ab^	1.33 ^a^	0.08	0.01
∑PUFA	24.53	27.23	28.17	2.15	0.28
n6/n3	24.01 ^a^	22.44 ^ab^	20.12 ^b^	1.19	0.046

^1^ n = 9 replicates per treatment; Means in a row with different letters are different significantly (*p* < 0.05); ^2^ PO = dietary palm oil; PO-RBO = The blend of dietary palm oil and rice bran oil; RBO = dietary rice bran oil.

## Data Availability

The datasets that were generated for this study are available on request to the corresponding author.
